# Shock Waves and Therapeutic Exercise in Greater Trochanteric Pain Syndrome: A Prospective Randomized Clinical Trial with Cross-Over

**DOI:** 10.3390/jpm13060976

**Published:** 2023-06-10

**Authors:** Angela Notarnicola, Ilaria Ladisa, Paola Lanzilotta, Davide Bizzoca, Ilaria Covelli, Francesco Paolo Bianchi, Giuseppe Maccagnano, Giacomo Farì, Biagio Moretti

**Affiliations:** 1 Orthopedics Unit, Department of Translational Biomedicine and Neuroscience “DiBraiN”, School of Medicine and Surgery, University of Bari, General Hospital, 70124 Bari, Italy; 2 Course of Motor and Sports Science, Department of Precision, Regenerative and Ionian Area Medicine, School of Medicine and Surgery, University of Bari, General Hospital, 70124 Bari, Italy; 3 UOSD Vertebral Surgery, AOU Consorziale Policlinico di Bari, 70124 Bari, Italy; 4 PhD Course in Public Health, Clinical Medicine and Oncology, Department DiMePre-J, University of Bari “Aldo Moro”, 70124 Bari, Italy; 5 Department of Interdisciplinary Medicine, School of Medicine and Surgery, University of Bari, 70124 Bari, Italy; 6 Orthopaedics Unit, Department of Clinical and Experimental Medicine, Faculty of Medicine and Surgery, University of Foggia, General Hospital, 71122 Foggia, Italy; 7 Department of Biological and Environmental Science and Technologies (Di.S.Te.B.A.), University of Salento, 73100 Lecce, Italy

**Keywords:** physiotherapy, shock waves, pain therapy, bursitis, hip, rehabilitation

## Abstract

This study aimed to verify the comparative effectiveness of shock wave therapy versus therapeutic exercise, including the possibility of combining both therapies, in patients who did not respond to the first treatment. A prospective randomized clinical trial was carried out, predicting the possibility of a cross-over between the two treatment options, with patients who did not respond to either treatment. Treatments were, respectively, eccentric therapeutic exercise consisting of 30 min sessions of stretching and strengthening exercises, 5 days a week for 4 weeks (Groups A and D) and Extracorporeal Shock Waves Therapy (ESWT) according to a protocol of three sessions, one per week, each of 2000 pulses at a 4 Hz frequency and administering an energy flux density (EFD) varying between 0.03 mJ/mm^2^ and 0.17 mJ/mm^2^ (Groups B and C). Patients were evaluated at baseline (T0), 2 (T1), 4 (T2) and 6 months (T3) after the last session, using the Numeric Rating Scale (NRS), Low Extremity Functional Scale (LEFS) and Roles and Maudsley Scale (RMS). The whole study population demonstrated a progressive clinical reduction in pain according to NRS, a recovery from disability according to LEFS and a perception of recovery according to RMS within 6 months, with no significant differences between the four protocols (exercise; ESWT; exercise + ESWT; and ESWT + exercise). Both therapies are therefore valid options in patients with trochanteritis; the combination of the two therapies could be evaluated for those patients who do not respond to the single treatment.

## 1. Introduction

The greater trochanteric pain syndrome (GTPS) is the cause of pain in 10–25% of patients with hip pain, with an incidence of approximately 1.8–5.6 patients per 1000 per year [1].

Traditionally, inflammation of one of the bursae in the trochanteric region has been recognized as the only cause of GTPS, and therefore treatments are aimed at resolving bursitis itself [2]. The complexity of hip biomechanics and the resulting problems of differential diagnoses make it difficult for clinicians to identify appropriate treatment protocols. However, conservative treatment is considered the primary approach; it includes rest, cryotherapy, biophysical therapy, kinesitherapy and possibly pharmacological treatment with non-steroidal anti-inflammatory drugs (NSAIDs) or local injections of corticosteroids and/or anesthetics or platelet-enriched plasma (PRP) [1,2]. Non-responder patients can be surgically managed [3].

Additional physical therapies such as shock waves (ESWT, Extracorporeal Shock Waves Therapy), laser therapy, diathermy and ultrasound play an important role [2]. 

Shock waves (SW) are defined as transient pressure oscillations that propagate in three dimensions and typically bring about a clear increase in pressure within a few nanoseconds [4]. There are very rapidly rising positive pressure impulses from 5 to 120 MPa in around 5 ns, followed by a decrease to negative pressure values of −20 MPa. It is commonly assumed that the ESWT induces the “destruction of calcific deposits” [5]. Previous authors suggest that shockwave therapy should be considered before surgery for chronic calcific tendinitis in patients after a minimum of 6 months of non-invasive treatment, with deposits greater than 1.5 cm and no radiological evidence of spontaneous disintegration. Recent studies confirm applications in tendinopathies also without calcification.

In chronic tendinopathies, the analgesic and anti-phlogistic effect of shock waves is proven, through tendon regeneration, hyperemia and overexpression of growth factors [6]. The beneficial biological and mechanical effects of focal shock waves are well demonstrated for tendon calcifications [7] and plantar fasciitis related to heel spurs [8], but this therapy has recently proved to also be very useful in treating other musculoskeletal diseases, such as muscle spasticity [9] and bone marrow oedema [10].

However, in the few studies regarding the application of acoustic waves in the treatment of GTPS, radial waves have been used to a greater extent [11,12]. While the latter is suitable for the treatment of large and superficial areas, focal ESWT induces a biological stimulus concentrated in-depth [13], and this could be an added advantage over radial waves in treating peritrochanteric soft tissues. In tendinopathies, it has also been shown that conservative treatment with eccentric exercise (EE), defined as slow movement progressing by adding load and not speed, is a valid therapeutic strategy and is superior to a generic exercise protocol [14]. Levels of evidence have varied between common tendinopathies, but there is evidence to suggest that eccentric exercise has a positive effect on clinical outcomes, such as reduced pain and improved joint function and patient satisfaction, compared to concentric exercise (CE), and that eccentric exercise may contribute to tendon repair processes by increasing collagen production [14]. Moreover, EE counteracts inflammation processes for bursae and tendons since it corrects biomechanical loads and promotes the production of anti-inflammatory interleukins [15,16]. 

The main goals of the therapeutic rehabilitative management of GTPS include strengthening of the gluteal muscles, stretching of the iliotibial band, load management with reduction of compressive forces on the GT and optimization of biomechanics.

This study aimed to verify the comparative effectiveness of focal extracorporeal shock wave therapy versus therapeutic exercise in terms of pain reduction and functional recovery in patients with GTPS, including the possibility of combining both therapies in patients who did not respond to the first treatment.

## 2. Materials and Methods

### 2.1. Study Design

A prospective, randomized, cross-over clinical trial with a single-blind assessment of outcomes (level of evidence 1-B) was conducted at the Bari General Hospital between November 2020 and September 2022. The study was approved by the Ethics Committee of the Bari General Hospital (resolution and protocol no. 1392-2020, 30 October 2020). 

### 2.2. Participants

Patients with GTPS who were referred to our orthopaedic outpatient service were consecutively recruited. Inclusion criteria were the following: diagnosis of gluteal tendinopathy for at least 3 months, confirmed by instrumental examination (ultrasound and/or magnetic resonance imaging); NRS ≥ 4; age between 18 and 80 years; and no corticosteroid injection or other conservative therapy in the previous 30 days. Exclusion criteria were partial or complete rupture of the gluteal tendons; the presence of other causes of hip pain such as dysplasia, deformity, severe hip osteoarthritis or low back pain; contraindications to shock waves (pacemakers, pregnancy, coagulation disorders, therapy with anticoagulants, history of neoplasia and epilepsy) or to standard physical activity (ischemic heart disease, arrhythmias, uncontrolled hypertension, heart failure and severe respiratory failure); peripheral vascular disorders (thrombophlebitis, thrombosis and lymphoedema); previous traumatic events in the last 3 months and surgical treatment of the lower limb or lumbar spine; neurological disorders; and chronic inflammatory diseases. All the recruited patients were informed and signed informed consent.

Forty-eight consecutive patients were considered, but four of them were not enrolled because they did not meet the inclusion/exclusion criteria; specifically, the following conditions emerged: multiple sclerosis, thrombocytopenia, cancer and a previous ipsilateral hip replacement. Thus, forty-four patients were initially randomized using STATA MP17 software and divided into 2 groups:

- Group A: Twenty-two patients undergoing eccentric therapeutic exercise according to the following protocol: 30 min per day, 5 days per week, for a total of 4 weeks of treatment and a total of 20 exercise sessions. Exercises included stretching for the pyriform muscle and the iliotibial tract, lifting the straight leg, squatting the band ll and strengthening the gluteus muscles. This rehabilitation protocol was carried out under the guidance of a therapist and exercises were adapted for each individual within a pain-free range.
- Group B: Twenty-two patients undergoing treatment with focused ESWT, using an electromagnetic ESWT generator (MINILITH SL1-0G, Storz Medical).

Patients who did not receive any benefit from the treatment at T1 (no reduction of at least two points in the NRS score) were crossed over to the other treatment (2 groups: C and D), according to the same type of sessions and duration of the individual groups, identifying the following 2 new groups:

- Group C: Patients who belonged to Group A were subsequently treated with focused ESWT, according to the same protocol as Group B.
- Group D: Patients previously in Group B were subsequently treated with therapeutic exercise, again according to the same type of sessions and duration as in Group A.

### 2.3. Intervention

**Figure 1:**
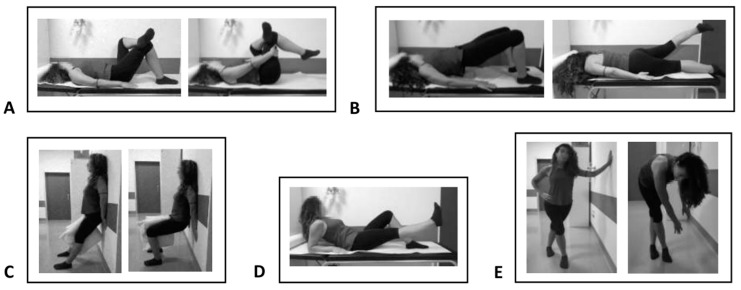
Eccentric therapeutic exercise: Piriformis muscle stretching (**A**); gluteal muscle stretching (**B**); wall squat with a ball (**C**); leg lift (**D**); and iliotibial band stretching (**E**).

Eccentric Physical Exercise: Patients belonging to Group A and patients belonging to Group D were subjected to eccentric therapeutic exercise for 5 days a week and a total of 4 weeks of treatment, thus for a total of 20 exercise sessions according to the scheme described above (Figure 1).

**Figure 2:**
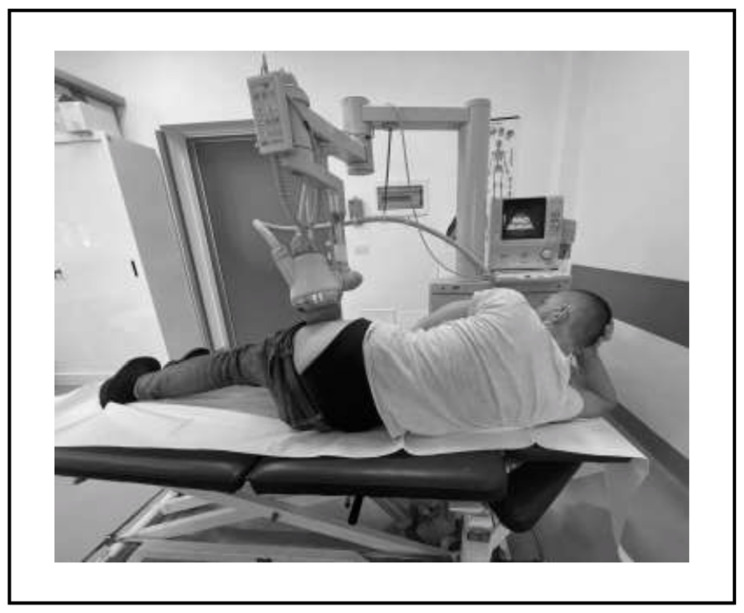
The patient is positioned on the side and the treatment probe is placed on the area to be treated, using an online ultrasound guide.

ESWT: Patients belonging to Group B and patients belonging to Group C were treated with focused shock waves, through the use of the electromagnetic ESWT delivery generator (MINILITH SL1-0G, Storz Medical). The patients were positioned on the treatment table in the lateral decubitus position and with ultrasound guidance. The treatment was delivered on the area of the greater trochanter and the enthesis of the gluteal muscle tendons, without local anesthesia and with ultrasound gel (Figure 2). The protocol included 3 sessions, 1 per week, according to the specific literature for other musculoskeletal pathologies and to the guidelines of the International Society for Shock Wave Treatment (ISMST) [14]. For each session, 2000 pulses were applied with a frequency of 4 Hz and an energy flux density (EFD) varying between 0.03 mJ/mm^2^ and 0.17 mJ/mm^2^, depending on the patient’s pain tolerance levels.

Patients were assessed at baseline (T0) through medical history, objective examination, imaging and validated scales, and at 2 (T1), 4 (T2) and 6 months (T3) after the last session through a re-evaluation of objective examination, imaging and the rating scales described below. The model was single-blind because the investigator performing the assessments and administering the scales did not know which treatment each patient had undergone.

### 2.4. Outcome Measures

The following rating scales were used:

- Numeric Pain Rating Scale (NRS), from 0 (no pain) to 10 (unbearable pain) points [17].
- Lower Extremity Functional Scale (LEFS), which is a valid patient-rated outcome measure for the measurement of lower extremity function; it quantifies the degree of difficulty in performing 20 types of activities of daily living, so patients are requested to select an answer for each activity (extreme difficulty or unable to perform activity = 0; no difficulty = 4), and the total amount ranges from 80 (very high function) to 0 (very low function) [18].
- Roles and Maudsley Scale (RMS), which measures the patient’s perception of improvement, from 1 (excellent result) to 4 (identical or worse symptoms than before treatment). This score is a categorical classification scale that has three dimensions: pain, movement and activity. It has been widely used for reporting the results of shock wave treatments [19,20].

### 2.5. Statistical Analysis

To estimate the sample size, a considered NRS value at enrollment (T0) of 8.0 for both groups was hypothesized, with an average value at the primary endpoint (T1) of 4.5 ± 2.0 in Group A and of 2.5 ± 1.5 in Group B (hypothesis of the researcher). The sample estimation was performed with the *t*-test, showing a significance level (alpha) of 0.025 and a test power of 85% that was set. A sample of 38 subjects was estimated; assuming a 15% loss at follow-up, the number of subjects to recruit is 44 (22 per group). This effect was selected as the smallest effect that would be important to detect, in the sense that any smaller effect would not be of clinical or substantive significance. Software Stata MP17 was used to calculate the sample size.

Randomization was performed with homogeneous randomization criteria for gender and age using a predefined program. The analysis was performed using Stata MP17 software. Continuous variables are expressed as mean ± standard deviation and range, and categorical variables as proportions. The normality of continuous variables was assessed using the Skewness and Kurtosis test and for those not normally distributed, where possible, a normalization model was constructed. Continuous variables were compared between groups using the one-way (parametric) ANOVA test, using Bonferroni correction to compare variables between individual groups. The ANOVA for repeated measures test was used to compare continuous variables between groups and detection times. The chi-square test or Fisher’s exact test was used to compare categorical variables between groups. Multivariate linear regression was used to assess the association between the difference in NRS and LEFS between T3 and T0 and for RMS between T3 and T1, using the group variable as determinant, adjusted for age, sex and BMI; the correlation coefficient was calculated, with the indicated 95% confidence interval (95% CI). A *p*-value < 0.05 was considered significant for all tests.

## 3. Results

**Figure 3:**
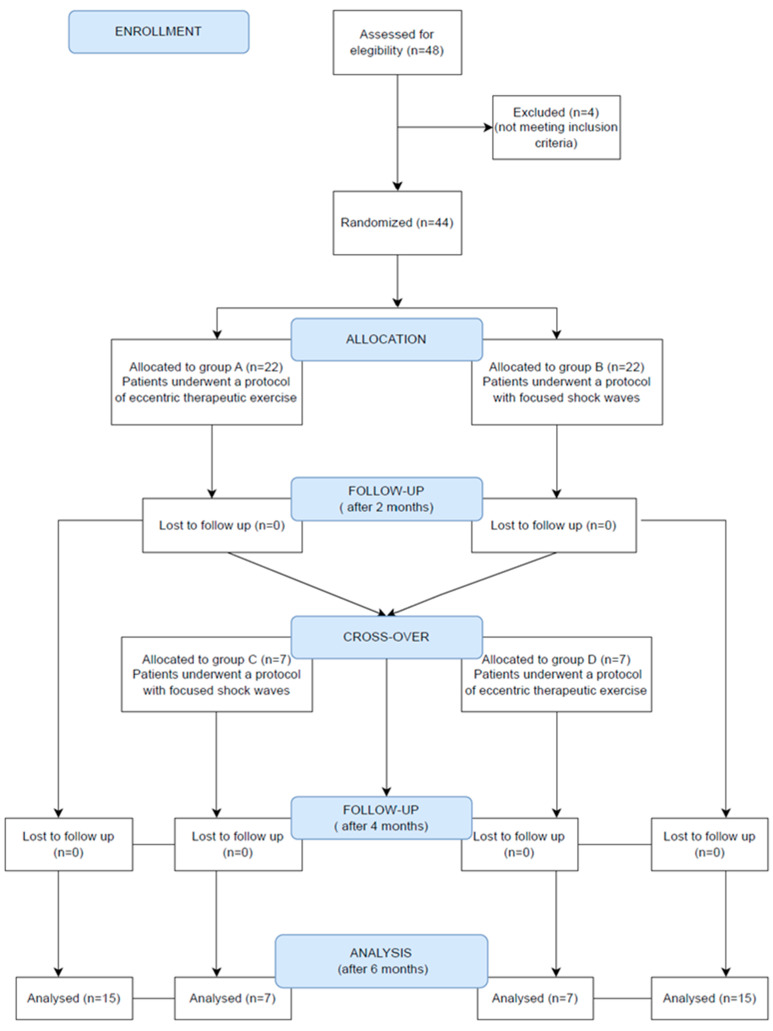
CONSORT diagram.

The study sample consisted of forty-four subjects, including thirty-six women (81.8%) and eight men (18.8%, Figure 3). The mean age of the patients recruited was 58.9 ± 10.4 years; the mean height was 164.0 ± 6.5 cm; the mean weight was 74.2 ± 10.4 kg; and the mean BMI was 27.3 ± 4.1 kg/cm^2^. Sample characteristics at the baseline are described in Table 1. The initial diagnoses according to MRI and ultrasound are described in Table 2.

In the study group, 15 (34.1%) subjects were treated with eccentric exercise (Group A), 15 (34.1%) were treated with shock waves (Group B), 7 (15.9%) were initially treated with eccentric exercise and at T1 underwent shock wave therapy (Group C) and 7 (15.9%) were initially treated with shock waves and at T1 underwent eccentric exercise (Group D).

Bonferroni correction did not show statistically significant differences between the individual groups concerning epidemiological characteristics (gender, age and BMI) (*p* > 0.05). In all four groups, there was a progressive reduction in pain (NRS), disability (LEFS) and perception of recovery (RMS) at the three study times (Table 3).

The ANOVA analysis for repeated measures showed a statistically significant difference in the comparison of NRS scores between times (*p* < 0.0001); no statistically significant differences were observed in the comparison between groups (*p* = 0.358) and in the interaction between times and groups (*p* = 0.511). The ANOVA analysis for repeated measures showed a statistically significant difference in the comparison of RMS between times (*p* < 0.0001), while no statistically significant differences were observed in the comparison between groups (*p* = 0.755) and in the interaction between times and groups (*p* = 0.964). The ANOVA analysis for repeated measures showed a statistically significant difference in the comparison of LEFS between times (*p* < 0.0001), while no statistically significant difference was observed in the comparison between groups (*p* = 0.207) and in the interaction between times and groups (*p* = 0.393).

The multivariate analysis highlighted a statistically significant inverse proportionality relationship between the RMS difference between T3 and T1 and age (coef. = −0.03; 95% CI = −0.04–0.01; *p* = 0.032) and a relationship at the limits of statistical significance between the difference between T3 and T0 of LEFS of Group B in comparison to Group A (coef. = 8.7; 95% CI = −0.01–17.4; *p* = 0.050); no further associations were observed between the outcomes and determinants under analysis (*p* > 0.05).

## 4. Discussion

This study demonstrates the validity and efficacy of the treatment strategy for GPTS of both treatments (EE and ESWT) in both single and combined modalities in patients not responding to a single treatment. In particular, concerning the combination of the two treatments, cross-over proved to be effective regardless of the temporal sequence in which the individual treatments followed each other. This combined treatment strategy allowed patients who had no benefit in terms of pain reduction after 2 months to recover promptly. However, although both treatments were effective, it was found that patients who received shock wave treatment alone had a more marked recovery from a functional point of view, as evidenced by a more marked improvement on the LEFS scale between T3 and T0. This suggests that shock wave treatment can guarantee a greater functional recovery if both methods are effective in pain management. Another finding from the statistical analysis was that age is a factor that can influence the subjective response to the effectiveness of the therapy performed. Hence, according to an inverse proportionality relationship, elderly subjects are generally less satisfied with the treatment.

On the other hand, the present study showed that patient non-responders to ESWT could also be refractory to EE.

These results are consistent with the available literature in which EE and ESWT appear among the recommended conservative alternatives in the therapeutic management of GTPS. Therapeutic exercise treatment appears to be effective, although there are not yet well-established programs, in terms of quantity speed and the number of contractions, and durations of treatment [20]. In particular, protocols usually include a combination of eccentric and concentric exercises, as there is no evidence in favor of one type over the other [21]. However, the beneficial effect of eccentric exercise in tendinopathies is known. The tendon, similar to the muscle, can adapt favorably to physical stress, including that of high eccentric loads. In particular, tendons become stronger as fibroblastic activity increases, with increased production of collagen. Macroscopic changes show a hypertrophic tendon, while microscopic adaptations are characterized by a thickening of collagen fibers and fibrils and an increase in tropocollagen crosslinks. Thus, the tendon fibers are optimally aligned to handle the high levels of stress transmitted from the muscle to the tendon [22].

Shock wave treatment has also progressively acquired a position of importance in the conservative treatment of tendinopathies [23,24] and therefore also in the management of GTPS [25]. ESWT is responsible for the “cavitation phenomenon”, which induces various biological-tissue responses such as angiogenesis, neuro-modulation, anti-inflammatory action and tissue regeneration [4]. The main biological effects are an improvement of vascularization, the release of growth factors, anti-inflammatory effects, advancement of axonal regeneration, pain relief and a muscle relaxant effect [26]. The anti-inflammatory effect, due to ESWT, is characterized by modulation of various molecular mechanisms such as endogenous nitric oxide synthesis, up-regulation of IL-6, IL-8 and IL-10, down-regulation of TNF-α and reduction of leukocyte infiltration [27]. The increase of tissue concentration of substance P and prostaglandin E2, verified a few hours after the application of SW, represents one of the main mechanisms responsible for the therapeutic effect and pain relief. During orthopaedic treatments, the accidental release of SW on epidermal nerve fibers has allowed the detection of transient nerve fiber degeneration, temporary reduction of certain peptides (protein gene product 9.5 and calcitonin gene-related peptide) involved in pain transmission and subsequent re-innervation reply within 2 weeks [28].

The first studies to evaluate the efficacy of shock waves in the conservative management of GTPS were those of Rompe and Furia using radial waves [11,12]. Rompe compared the use of radial waves with home exercise programs and local corticosteroid injections, showing a greater pain benefit for corticosteroids only initially at 1 month, while shock waves and home exercise programs were superior in the medium and long term. Furia also showed short- and long-term superiority of radial waves over a control group treated with conservative modalities, such as rest, cryotherapy, anti-inflammatory drugs, iontophoresis, stretching exercises, gluteal muscle and tensor fascia lata strengthening and local corticosteroid and anesthetic injections. Heaver et al.’s study stated that focal shock wave treatment is better in the long-term results compared to corticosteroid injections in GTPS [25].

In a recent retrospective study, Seo et al. showed the efficacy of ESWT (with an electro-hydraulic generator, EFD = 0.10 mJ/mm^2^) to relieve pain in patients with GTPS documented by MRI, with effects being reduced in the long term [13]. Follow-up was carried out by telephone at least 4 months after shock wave treatment but with an average of 27 months after treatment. Carlisi et al. showed, in a randomized clinical trial, that ESWT with the piezoelectric generator is more effective than ultrasound therapy in reducing pain in the short (2 months) and medium (6 months) term in the management of GTPS, while no differences were observed from a functional point of view with the LEFS scale analysis. Vulpiani and Ramon’s 2020 multicenter study is the only one that, similar to ours, evaluated the effectiveness of ESWT, using an electromagnetic generator for the treatment of GTPS vs. a control group treated with the lowest energy level provided by the device [29]. Both groups performed a therapeutic exercise protocol at home for 24 weeks; regarding the ESWT protocol used, three sessions with 2000 pulses were planned, as in our case, but constant energy was applied. The best results were found in patients exposed to higher energy levels. 

Comparing our work with the present literature [30], our study is the only one with a cross-over design that allowed us to evaluate the treatments’ effectiveness in purity and combination. 

The combination of the two treatments could represent a new and effective therapeutic option in cases of GTPS refractory to single treatments, expanding the range of conservative therapies and further limiting the use of surgical solutions.

The main weakness of our study is the small sample size. The second limitation is the lack of instrumental examinations at follow-up and monitoring with a baropodometric examination or gait analysis. The main strength of the study is that it provided for the possibility of a cross-over of the two treatments, allowing non-responsive patients to integrate the treatment pathway with the other treatment method.

## 5. Conclusions

In conclusion, EE and ESWT treatments performed either alone or in combination are effective in the therapeutic management of GTPS, both in terms of pain reduction and functional recovery, in all timings analyzed. The combination, irrespective of the time sequence, is successful in the recovery of patients unresponsive to a single treatment and is, therefore, to be considered a valid treatment strategy. As there is currently no univocal protocol for the management of GTPS, further studies are needed to confirm the long-term effectiveness of the treatments and to create increasingly standardized treatment protocols.

## Figures and Tables

**Table 1:** Sample size characteristics at the baseline.

	Group A	Group B	Group C	Group D	Total	*p*-Value
Age; average ± SD (range)	59.1 ± 9.6 (37–69)	59.5 ± 7.7 (49–73)	64.0 ± 12.7 (37–74)	52.1 ± 13.1 (31–74)	58.9 ± 10.4 (31–74)	0.196
Female; *n* (%)	12 (80.0)	13 (86.7)	4 (57.1)	7 (100.0)	36 (81.8)	0.245
BMI; average ± SD (range)	28.7 ± 6.0 (19.8–39.3)	27.5 ± 2.4 (24.3–33.0)	26.2 ± 2.3 (22.0–28.8)	25.0 ± 2.5 (20.8–28.0)	27.3 ± 4.1 (19.8–39.3)	0.218

**Table 2:** MRI and ultrasound diagnoses per group.

Diagnoses	Group A (Number of Patients)	Group B (Number of Patients)	Group C (Number of Patients)	Group D (Number of Patients)
Gluteus bursitis	9	9	2	3
Medius and minimus gluteus tendinopathies	9	9	3	2
Medius and minimus gluteus calcific tendinopathies	4	4	2	2

**Table 3:** Mean ± SD (range) of Numeric Rating Scale (NRS), Low Extremity Functional Scale (LEFS) and Roles and Maudsley Scale (RMS) by group and detection time.

Time	Group A (*n* = 15)	Group B (*n* = 15)	Group C (*n* = 7)	Group D (*n* = 7)	Total (*n* = 44)	Comparison between Groups	Comparison between Times	Interaction between Groups and Times
NRS								
T0	7.7 ± 1.4 (4–9)	8.3 ± 0.9 (7–10)	7.0 ± 1.2 (5–8)	8.3 ± 1.1 (7–10)	7.9 ± 1.2 (4–10)	0.358	<0.0001	0.511
T1	5.5 ± 1.6 (1–7)	5.4 ± 2.0 (1–7)	5.1 ± 2.0 (1–10)	6.9 ± 2.9 (1–10)	5.6 ± 2.0 (1–10)			
T2	5.0 ± 2.3 (1–8)	4.5 ± 2.0 (1–7)	4.3 ± 1.8 (1–6)	5.9 ± 2.7 (1–10)	4.8 ± 2.2 (1–10)			
T3	4.3 ± 2.6 (1–9)	3.9 ± 1.8 (1–6)	3.7 ± 1.6 (1–6)	5.3 ± 2.4 (1–9)	4.2 ± 2.1 (1–9)			
LEFS								
T0	48.7 ± 12.3 (24–76)	32.8 ± 13.8 (17–66)	40.4 ± 7.8 (32–53)	36.6 ± 8.9 (24–46)	40.0 ± 13.3 (17–76)	0.207	<0.0001	0.393
T1	53.8 ± 12.2 (24–76)	42.9 ± 19.3 (21–80)	48.7 ± 9.8 (40–66)	42.4 ± 17.4 (25–76)	47.5 ± 15.9 (21–80)			
T2	55.9 ± 15.5 (22–74)	48.8 ± 17.7 (21–80)	53.1 ± 14.4 (30–70)	48.6 ± 19.6 (28–77)	51.9 ± 16.5 (21–80)			
T3	59.0 ± 15.3 (28–75)	52.3 ± 15.1 (30–80)	55.0 ± 14.3 (30–72)	49.4 ± 19.1 (30–77)	54.5 ± 15.6 (28–80)			
RMS								
T1	2.7 ± 0.6 (2–4)	2.5 ± 0.8 (1–4)	2.6 ± 0.5 (2–3)	2.7 ± 0.8 (1–3)	2.6 ± 0.7 (1–4)	0.755	<0.0001	0.964
T2	2.3 ± 0.8 (1–4)	2.1 ± 0.7 (1–3)	2.1 ± 0.7 (1–4)	2.4 ± 1.0 (1–3)	2.2 ± 0.8 (1–4)			
T3	2.1 ± 0.9 (1–4)	2.0 ± 0.8 (1–3)	2.1 ± 0.7 (1–3)	2.4 ± 1.0 (1–3)	2.1 ± 0.8 (1–4)			

*n* = number of patients for each group at the baseline.

## Data Availability

The data that support the findings of this study are available from the corresponding author upon reasonable request.
